# Fukushima Nuclear Power Station: What Happened? Why All Health Care Professionals Need Radiation Training

**DOI:** 10.1007/s00266-011-9852-3

**Published:** 2012-03-10

**Authors:** Kazuko Ono

**Affiliations:** Kyoto Medical College of Science, Kyoto, Japan

## The Fukushima Accident

On 11 March 2011, a magnitude 9 earthquake occurred beneath the seabed about 130 km off the northeastern coast of the main island of Japan. The Fukushima Dai-idhi nuclear power station (FNP-I) with its six reactors is operated by the Tokyo Electric Power Company (TEPCO) on the coast about 200 km southwest of the epicenter. The location of FNP-I is about 230 km north of Tokyo.

**Fig. 1:**
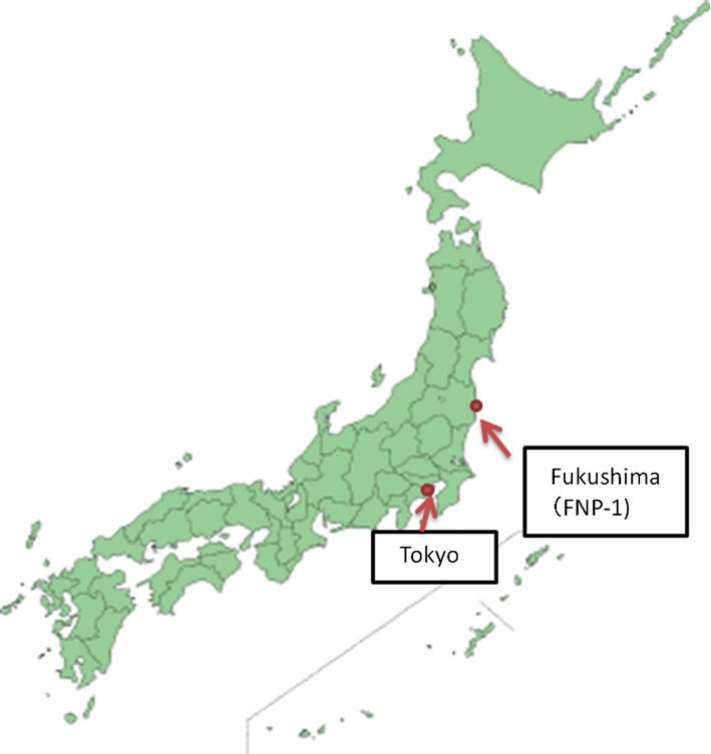
Map of Japan. The Fukushima Dai-ichi nuclear power station is on the coast about 230 km from Tokyo

When the earthquake hit FNP-I, three of its six reactors were in operation. Although they were shaken beyond the magnitude assumed in the design, the safety system successfully shut down all the reactors automatically. But a pylon for the power line for FNP-I collapsed, cutting off the supply of electricity to FNP-I. About 40 min later, the huge tsunami that overwhelmed the sea wall crashed into the turbine buildings that contained the diesel generators for the emergency power supply. Because all electric power was lost, the cooling systems of the reactors were paralyzed.

As the loss of coolant accident (LOCA) proceeded in reactors 1 to 3, the suppression chamber of reactor 2 fractured in the early morning of 15 March, causing a major release of radioactive materials. Except for noble gases, the principal components of the released radioactive materials were volatile [i.e., radioiodine (iodine 131) and radiocesium (cesium 134 and 137)]. Although slight amounts of some radioactive materials with relatively low boiling points such as radiotellurium (tellurium 132) were observed, there was no radiostrontium (strontium 90) in the plume (basic information can be found at Nuclear Safety Commission http://www.nsc.go.jp/NSCenglish).

## What Action was Taken by the Japanese Government?

The Japanese government ordered the inhabitants around FNP-I station to evacuate. The radius of the evacuation zone was expanded from 2 km on 11 March to 20 km on 12 March, and this early evacuation of some 20,000 inhabitants had been almost completed by 15 March. The evacuees younger than 40 years were administered stable iodine (potassium iodine) on 16 March to protect the thyroid from radioiodine uptake. People living in the area 20 to 30 km from FNP-I were ordered indoors on 15 March and advised to evacuate voluntarily on 25 March. The area within 20 km from FNP-I was assigned to the strictly controlled zone on 22 April to prohibit entrance.

Dose-rate monitoring in and around the 30-km zone on 15 March showed that several regions outside the zone had a higher dose rate than 50 microsieverts per hour (μSv/h)^1^ . These regions extend to more than 10 km toward the northwest, and the Iitate village of some 6,000 inhabitants was affected in particular. The dose rate exceeded 150 μSv/h at Nagadoro, the southernmost area of the village (Fig. 1).

The government assigned Iitate village and four other areas to the planned evacuation area and ordered on 22 April that all inhabitants should leave by the end of May. Although the radioiodine had decayed out in August 2011, the dose rate in the planned evacuation area still was higher than 10 μSv/h (the value monitored by the government at the reference point in the village) due to radiocesium contamination.

The government declared values of maximum permissible radioactive concentration in foods and drinks on 17 March. The values were based on a scenario in which those maximally exposed to contaminated foods and drinks would receive annual doses up to 5 mSv. Radioiodine-contaminated-squeezed milk beyond the limit was for the first time reported on the very day, and a report on spinach followed the next day.

The risk of thyroid cancer by taking in radioiodine released from the FNP-I accident was one major concern of the public in the early stage because many people had known of the increase in pediatric thyroid cancer after the Chernobyl accident. Monitoring of I-131 gamma rays from the thyroid was carried out for more than 1,080 children from severely affected areas, with no child showing a higher dose rate than the screening level of 0.2 μSv/h (press release from Nuclear Safety Commission).

In June, the Japanese government decided to measure radioactive concentrations in breast milk. We measured 95 subjects and 12 control subjects. No radioiodine was detected, but radiocesium was detected in seven of the subjects (2–13 Bq/kg). This low concentration of radiocesium, however, will not cause any risk for babies because about 60 Bq/kg of radiopotassium, which has a similar chemical property and emits more energetic beta-rays and gamma-rays, naturally exists in their bodies (press release was only in Japanese).

## What Action was Taken at the Site?

Hundreds of workers were struggling around the reactors to stabilize the situation. Even 2 months after the accident, they had to work in very poor working conditions. They had to sleep on the floor and had to take reserved emergency rations that could be contaminated.

The dose limit for the occupational radiation worker in Japan is defined, based on the The International Commission on Radiological Protection (ICRP) Recommendations, as 20 mSv per year averaged over 5-year periods, provided the effective dose for any single year does not exceed 50 mSv. Moreover, the government defined the dose limit for male emergency workers at the FNP-I site as 250 mSv (throughout a man’s lifetime). The average lifetime risk of cancer incidence for a worker receiving a whole-body exposure of approximately 250 mSv is nominally assessed as 1 to 2% above the natural occurrence of about 40% for the general Japanese population. Currently, a few workers have received doses exceeding 250 mSv.

## The Current Situation in Japan

As of early August, all three damaged FNP-I reactors were cooled to core temperatures below 110°C at atmospheric pressure. The government has declared that the risk of another accident is quite small. Many decontamination programs are going to begin. The International Nuclear and Radiological Event Scale (INES) designed for communication to the public on the severity of events assessed the FNP-I accident as level 7 (a major accident). However, the amount of radioactive material released into the atmosphere was about 10% that in the Chernobyl accident. Successive dose-rate monitoring has been conducted by the government together with universities (http://www.mext.go.jp/component/a_menu/other/detail/__icsFiles/afieldfile/2011/08/04/1309290_080410u.pdf), and the dose-rate in Tokyo/Osaka/Kyoto is the same as that measured before the accident. The monitored radioactive concentration levels in drinking water samples also have been reported. None of them showed detectable contamination with I-131, Cs-134, or Cs-137. Based on the scenario used for deriving the maximum radioactive concentration in food (500 Bq/kg), an individual must eat at least 154 kg of maximally contaminated beef or 2,000 maximally contaminated rice balls to receive 1 mSv.

Misunderstandings about “hot spots” also are a problem. Because the radioactive fallout can be drawn by rain, currently observed dose rates vary from place to place. Although the dose rates around places where rain water collects contamination are locally elevated, they still are far below the level that could result in an annual dose of 1 mSv.

## Medical Problems in the Affected Areas

Information obtained from the Internet, however, is not always correct and often fragmented or distorted. Such inadequate information often is more resonant with people’s anxiety.

Many pregnant women are anxious about the effects of ionizing radiation on their fetus. They are worrying about congenital malformations and pediatric cancers, although the prenatal doses their fetuses received in the accident remain far below the threshold for inducing malformation.

Although patients often ask medical doctors about their anxiety, many physicians unfortunately cannot correctly answer because they do not have proper knowledge and understanding about the effects of low-level exposure to ionizing radiation on human health. Even worse, some doctors estimate the possibility of future cancer incidence using a so-called linear nonthreshold (LNT) mode and announce the figure on the Internet. They ignore the statement of the ICRP about the retrospective use of the LNT model in the 2007 Recommendations and will not pay attention to the comments that disagree with their belief. The author is afraid that such misuses of the LNT model might spoil future effective use of radiation in medicine.

The experience after the FNP-I accident taught us the necessity of better training for medical and paramedical staff concerning the effects of ionizing radiation on human health and concerning the system of radiation protection. Such training is especially important for those working in local hospitals because most members of the public believe that medical workers have sufficient knowledge on the issue of radiation and radioactivity and their influence on the health. When a radiologic emergency situation occurs, they are expected to answer questions on the degree of danger and provide practical and useful advice.

Members of the Radiation Protection Board of the Japan Radiological Society were involved in the medical support of the Fukushima population. We will continue efforts to dissolve irresponsible rumors about the effects of radiation that may harm people or hinder the positive uses of radiation in medicine.

## Footnotes

1 Sievert is the special unit name of the “effective dose” and the unit symbol, Sv, is assigned for the unit. The effective dose is a radiation quantity used for protection purposes.

